# Diagnosis and treatment of chronic constipation – a European perspective

**DOI:** 10.1111/j.1365-2982.2011.01709.x

**Published:** 2011-03-16

**Authors:** J Tack, S Müller-Lissner, V Stanghellini, G Boeckxstaens, M A Kamm, M Simren, J-P Galmiche, M Fried

**Affiliations:** * Division of Gastroenterology, University Hospital LeuvenLeuven, Belgium; † Department of Internal MedicinePark-Klinik Weissensee, Berlin, Germany; ‡ Department of Clinical Medicine, University of BolognaBologna, Italy; § Departments of Medicine and Gastroenterology, St Vincent's HospitalMelbourne, Australia and Imperial CollegeLondon, UK; ¶ Department of Internal Medicine, Sahlgrenska University HospitalGöteborg, Sweden; ** Department of Liver and Gastroenterology, Institute of Diseases of the Digestive System NantesCHU Nantes, France; †† Division of Gastroenterology and Hepatology, University Hospital ZurichZurich, Switzerland

**Keywords:** algorithm, constipation, dissatisfaction, prokinetic, prucalopride

## Abstract

**Background:**

Although constipation can be a chronic and severe problem, it is largely treated empirically. Evidence for the efficacy of some of the older laxatives from well-designed trials is limited. Patients often report high levels of dissatisfaction with their treatment, which is attributed to a lack of efficacy or unpleasant side-effects. Management guidelines and recommendations are limited and are not sufficiently current to include treatments that became available more recently, such as prokinetic agents in Europe.

**Purpose:**

We present an overview of the pathophysiology, diagnosis, current management and available guidelines for the treatment of chronic constipation, and include recent data on the efficacy and potential clinical use of the more newly available therapeutic agents. Based on published algorithms and guidelines on the management of chronic constipation, secondary pathologies and causes are first excluded and then diet, lifestyle, and, if available, behavioral measures adopted. If these fail, bulk-forming, osmotic, and stimulant laxatives can be used. If symptoms are not satisfactorily resolved, a prokinetic agent such as prucalopride can be prescribed. Biofeedback is recommended as a treatment for chronic constipation in patients with disordered defecation. Surgery should only be considered once all other treatment options have been exhausted.

## Introduction

Constipation is very common and many or most people are affected at some time in their life. However, for up to a quarter of the population it is more than a minor annoyance; for them, constipation can be chronic, sometimes severe, and has a significant, even debilitating, effect on their quality of life.^1^–^3^ Many patients suffer in silence and try to self-medicate, while for those who do seek medical help, treatments can be unsatisfactory.^2^,^4^ Nonetheless, in the authors’ experience, most patients can be helped with the right treatment approach. There are few detailed guidelines and recommendations available for the management of chronic constipation, and these do not always include more recently available treatments. Therefore, this article provides a summary of the condition from a European perspective, reviewing the pathophysiology, diagnosis and current management of constipation, including more recently available therapeutic agents.

It is estimated that constipation affects between 2% and 27% of the population, depending on the definition used,^5^,^6^ with 12% of people worldwide reporting self-defined constipation.^7^ However, the prevalence of constipation can be underestimated, as at least 65% of patients suffering from constipation do not seek immediate medical advice, but use over-the-counter (OTC) laxatives instead.^2^,^5^

Constipation may be severe and chronic; in a survey of over 10 000 individuals in the US, 14.7% met the criteria for constipation and 45% of these respondents reported having the condition for 5 years or more.^6^ Some experience weeks without a bowel movement and suffer from bloating, straining, defecation urge with an inability to evacuate, and painful evacuation.^2^,^3^ Daily activities and ability to work can be compromised, e.g., by discomfort, the need to be near a toilet, and by the length of time it takes to defecate. Measures of general health, social functioning, and mental health are significantly impaired compared with healthy individuals and levels are comparable with other conditions such as osteoarthritis, rheumatoid arthritis, chronic allergies and diabetes.^1^

In a web-based survey of approximately 4600 US respondents reporting one or more symptoms of constipation, only one in four reported seeking treatment from a doctor in the previous year.^2^ Those who do seek medical treatment are often not effectively treated, and only between one-quarter to two-thirds of patients with chronic constipation are satisfied with their laxative treatment.^2^,^4^ In a European survey of 744 patients with chronic constipation, almost half were using alternative treatments (homeopathy, massage and acupuncture) and one in three were not taking any medication.^4^ Nearly 90% of respondents expressed interest in new therapies.

## Pathophysiology

Constipation may be primary (idiopathic) or secondary to other factors (Table 1).^8^

**Table 1:** Causes of secondary constipation^8^

Cause	Example
Organic	Colorectal cancer, extra-intestinal mass, postinflammatory, ischemic or surgical stenosis
Endocrine or metabolic	Diabetes mellitus, hypothyroidism, hypercalcemia, porphyria, chronic renal insufficiency, panhypopituitarism, pregnancy
Neurological	Spinal cord injury, Parkinson's disease, paraplegia, multiple sclerosis, autonomic neuropathy, Hirschsprung's disease, chronic intestinal pseudo-obstruction
Myogenic	Myotonic dystrophy, dermatomyositis, scleroderma, amyloidosis, chronic intestinal pseudo-obstruction
Anorectal	Anal fissure, anal strictures, inflammatory bowel disease, proctitis
Drugs	Opiates, antihypertensive agents, tricyclic antidepressants, iron preparations, anti-epileptic drugs, anti-Parkinsonian agents (anticholinergic or dopaminergic)
Diet or lifestyle	Low fiber diet, dehydration, inactive lifestyle

**Figure 1:**
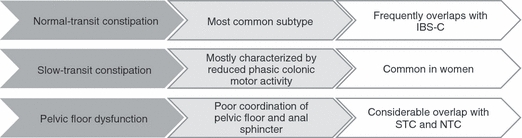
Types of constipation.Primary (idiopathic) constipation can be conceptually categorized into three main types: normal-transit, slow-transit and pelvic floor dysfunction. IBS-C, constipation predominant irritable bowel syndrome; STC, slow-transit constipation.^8^

Several sub-types of primary constipation are recognized, however, patients can display symptoms consistent with those from several sub-types. Constipation may be slow-transit (prolonged delay in passage of stool through the colon), or normal-transit. With slow and normal-transit times, there may be functional obstruction in the form of dysfunction of pelvic floor and anal sphincter muscles (anismus), leading to difficulty in expelling stools from the anorectum, or there can be no identifiable pathology with normal-transit constipation (Fig. 1).

### Normal-transit constipation

Normal-transit constipation is probably the most common form of constipation seen by general clinicians, although this has not formally been studied.^8^,^9^ Stool traverses at a normal rate through the colon and stool frequency may be normal, but patients feel constipated.^10^ This group of patients report difficulty with evacuation, bloating, abdominal pain or discomfort and hard stools; however, reduced colonic transit cannot be confirmed.^11^ Constipation based on a patient's report of frequency of bowel movements may be underestimated without the use of a stool diary.^10^

On investigation, some patients in this group may have increased rectal compliance, reduced rectal sensation or both.^12^ A significant overlap exists between this subgroup of constipation and the irritable bowel syndrome (IBS) subgroup.

### Slow-transit constipation

Approximately half of patients with symptoms refractory to supplementary fiber have a prolonged intestinal transit time.^12^ This group of patients has been found to have significant impairment of propulsive colonic motor activity,^13^ and significantly diminished colonic responses following a meal and on awakening in the morning;^10^ however, diurnal rhythm is usually preserved.^14^ In some patients, slow transit may be the result of an underlying defecation disorder, as colon transit times can become normalized following treatment (e.g., using biofeedback) for the underlying problem.^15^

The impaired motility in slow-transit constipation may be due to a number of factors. Propagating pressure waves in the colon, known as high amplitude propagated contractions (HAPCs), are strong contractions that begin at variable points in the colon and propagate towards the rectum. Several studies have shown that HAPCs are significantly decreased in constipated patients.^14^,^16^ A potential basis for these impairments are abnormalities of the myenteric plexus, characterized by a reduction in the number of argyrophilic neurons and axons, and an increase in the number of variably sized nuclei within ganglia.^17^ Interstitial cells of Cajal may also be important since their volume is significantly reduced in patients with slow-transit constipation.^18^ Interstitial cells of Cajal are required for generating the smooth muscle electrical slow wave, thus determining its contractile activity. In the absence of an electrical slow wave, contractile activity is reduced and irregular, resulting in decreased intestinal transit.^18^ Alternatively, the excitatory extrinsic nervous input to the bowel may be diminished, or extrinsic inhibitory activity to the bowel may be enhanced.

Acetylcholine is a neurotransmitter at neuro-neuronal and neuro-effector synaptic junctions within the enteric nervous system (ENS), and release of acetylcholine from enteric postsynaptic cholinergic neurons is the primary stimulant for spontaneous contractile activity of colonic smooth muscle.^19^ Compared with healthy subjects, constipated patients display an impaired colonic motor response to cholinergic stimulation in the descending colon.^19^ Serotonin [5-hydroxytryptamine (5-HT)], released from enterochromaffin cells in the gastrointestinal (GI) tract, stimulates neurons of the myenteric plexus to initiate peristaltic and secretory reflexes. While it is clear that 5-HT signaling is dysfunctional in intestinal motility disorders,^20^ it is not yet apparent whether this is underpinned by changes in the availability of 5-HT *per se,* or whether 5-HT re-uptake mechanisms, receptor density and/or function are diminished.^21^

It has been suggested, but not anatomically proven, that neuronal damage arising from neurodegeneration, or from damage during pelvic surgery or childbirth, reduces colonic motility and may underlie certain cases of idiopathic slow-transit constipation.^22^

Although the relationship between sex hormones and chronic constipation is not clear, a decreased level of ovarian and adrenal steroid hormones has been reported in association with constipation.^23^ Furthermore, one *in vitro* study proposed a mechanism for slow-transit constipation where the over-expression of progesterone receptors can down-regulate contractile G-proteins and up-regulate inhibitory G-proteins in colonic circular muscle cells.^24^

### Defecation disorders

A number of patients with chronic constipation display a difficulty in expelling stools from the rectum. This failure may be due to impaired rectal contraction, paradoxical anal contraction, or inadequate anal relaxation.^25^ Lack of coordination, or dyssynergia, of the muscles involved in defecation is thought to be the most likely cause,^26^ but a high proportion of patients may also show impaired rectal sensation.^25^ Structural abnormalities are less common but include rectal prolapse and/or intussusceptions, rectocele (a herniation, usually of the anterior rectal wall towards the vagina), and excessive perineal descent. In many patients, pelvic floor dysfunction may contribute to constipation with or without delayed transit, and as a consequence, biofeedback therapy has been shown to be beneficial in recent controlled trials.^27^–^29^

Many constipated patients show reduced sensitivity to slow rectal distension, suggesting that there may be diminished sensory innervation to the rectum and sigmoid colon. In addition to a reduced urge to defecate, this may indicate an imbalance between sympathetic and parasympathetic influences in some constipated patients, associated with decreased propulsive motor activity and tone.^12^

## Diagnosis of chronic constipation

The duration and characteristics of the patient's symptoms must be assessed to distinguish chronic from transient constipation. Transient constipation is easily recognized by history, indicating constipation started at a time of change in dietary habits, mobility or lifestyle. Secondary constipation, as a consequence of other factors (Table 1), should be identified and treated accordingly.

### Diagnostic resources

#### Rome III criteria

The Rome III classification system is widely recognized as the only standardized symptom-based diagnostic criteria for functional GI disorders (FGIDs), including chronic constipation (Table 2).^30^ Other definitions of chronic constipation are consistent with the Rome III criteria but are less quantitative and more subjective.^31^,^32^ Although clinicians are aware of the Rome criteria, these are used principally for research purposes and are not widely applied in clinical practice, with the possible exception of IBS.^33^ However, the Rome Foundation diagnostic algorithm project has recently published a new set of clinical algorithms for FGIDs, including chronic constipation, which make active use of the Rome criteria for diagnostic and therapeutic management (discussed in section entitled Review of currently available guidelines, recommendations and algorithms).

**Table 2:** Rome III criteria for chronic constipation^30^

Criteria fulfilled for the last 3 months and symptom onset at least 6 months prior to diagnosis
Presence of ≥2 of the following symptoms:
• Lumpy or hard stools in ≥25% of defecations
• Straining during ≥25% of defecations
• Sensation of incomplete evacuation for ≥25% of defecations
• Sensation of anorectal obstruction/blockage for ≥25% of defecations
• Manual maneuvers to facilitate ≥25% of defecations (digital manipulations, pelvic floor support)
• <3 evacuations per week
Loose stools rarely present without the use of laxatives
Insufficient criteria for irritable bowel syndrome

#### Bristol Stool Form Scale

The Bristol Stool Form Scale (BSFS) 34 is a useful visual aid that was designed to assist in the evaluation of patients with constipation. Using simple visual descriptors, it illustrates the common stool forms and consistency on a 7-point scale. It has been validated in a number of studies and has been found to be easily understood by patients, enabling them to recognize and thus classify the stool type that most closely represents their own experience. The form of the stool depends on the time that it spends in the colon; therefore, the BSFS is a quick and reliable indicator of transit time. It is particularly useful in patients with self-reported constipation who do not have infrequent bowel movements, to establish that hard or lumpy stools are, indeed, present.

### Diagnostic evaluation

Routine extensive diagnostic and physiological testing is not recommended for chronic constipation.^32^,^35^ However, in those not responding to initial treatment, three main physiological tests can be used to assess anorectal disorders (e.g., dyssynergic defecation): anorectal manometry,^25^,^26^,^36^ the balloon expulsion test 36 and colon transit studies.^36^,^37^ If necessary, colonoscopy can be used to detect the presence of lesions such as rectal ulcers, inflammation or malignancy, and radiography of the abdomen can provide evidence for an excessive amount of stool in the colon. Barium enema plays a limited role today but can identify megacolon and megarectum. Anorectal manometry and histology of the nerve plexuses can be used to confirm Hirschsprung's disease.

As discussed earlier, constipation may arise secondary to a variety of factors (Table 1).^8^ Secondary causes of constipation are recognized by careful history taking and clinical examination, including a thorough rectal examination (e.g., the 10-step approach described by Talley).^38^ Colonoscopy is recommended in case of alarm symptoms and in those over 50 to screen for colorectal cancer.^8^,^39^ Alarm features suggestive of a serious GI disorder requiring further investigation include:^40^

1. Weight loss.
2. Blood in the stool.
3. Anemia.
4. Sudden change in bowel habit after the age of 50.
5. Significant abdominal pain.
6. Family history of colon cancer or inflammatory bowel disease.

Hence, routine diagnostic testing is generally not recommended for chronic constipation. However, if there is suspicion of metabolic causes, these can be investigated with a complete blood cell count, biochemical profile, serum calcium, glucose levels and thyroid function tests. If these give rise to suspicion, serum protein electrophoresis, urine porphyrins, serum parathyroid hormone and serum cortisol levels can be considered, but this will only rarely be indicated.^10^

## Treatment options

### Current treatment options

Current laxatives aid defecation by decreasing stool consistency (softening) and/or artificially or indirectly stimulating colon motility, via one or more of a number of mechanisms (Table 3).^41^,^42^

**Table 3:** Drugs commonly used in the treatment of constipation^41^,^42^

Laxative type	Examples	Proposed mode of action	Potential limitations
Dietary fiber/bulking agents	Wheat bran Psyllium seed husk Methylcellulose	Luminal water binding increases stool bulk and reduces consistency	Flatulence and abdominal distension Stool impaction (rarely) Not recommended in frail, immobile, or palliative care patients
Osmotic laxatives			
Undigestible disaccharides and sugar alcohols	Lactulose Sorbitol	Luminal water binding by creating an osmotic gradient	Bloating, flatulence
Synthetic macromolecules	PEG Polycarbophil	Luminal water binding	Bloating
Salinic laxatives	Magnesium hydroxide (e.g., milk of magnesia) Magnesium citrate Magnesium sulfate Sodium phosfate	Luminal water binding Increases fluid excretion	Electrolyte imbalance (must be used with caution in patients with compromised renal or cardiac function)
Stimulant laxatives			
Diphenylmethane derivatives	Bisacodyl, sodium picosulfate	Act locally to stimulate colonic motility, decrease water absorption from large intestine	Abdominal discomfort and cramps
Anthraquinones	Senna, aloe, cascara	Act locally to stimulate colonic motility, decrease water absorption from large intestine	Abdominal discomfort and cramps

Well-designed, placebo-controlled, blinded, clinical trials of older laxatives are sparse. Although many trials report improvements in the number of bowel movements per week and some report improvements of certain symptoms, many studies are small and lack comprehensive clinically relevant treatment endpoints. Similarly, there is a lack of head-to-head comparisons; hence, there is a lack of evidence to determine whether one laxative class is superior to another. It is also largely unknown if laxative treatments address the impaired quality of life observed in patients with chronic constipation, as most studies have failed to assess quality of life measures. Indeed, for some patients, laxatives can worsen certain symptoms, such as bloating and flatulence.^43^

Undigestible fibers attract water, which leads to a larger and softer fecal mass. Systematic reviews of older studies indicate that fiber increases the number of bowel movements, but the quality of these studies is inconsistent and the treatment duration was usually limited to 4 weeks or less.^42^ It has been shown that patients with slow transit and/or impaired defecation are unlikely to respond to fiber.^44^

Most comparative data suggest that lactulose and polyethylene glycol (PEG) have similar efficacy, but with lower incidence of vomiting and flatulence associated with the latter.^45^,^46^ PEG provides well-tolerated and effective relief in constipated patients.^46^,^47^ In a 6-month placebo-controlled study, 304 patients with chronic constipation received either 17 g PEG or placebo. Fifty-two percent of PEG-treated patients compared with 11% of placebo-treated patients (*P*< 0.001) were successfully relieved from constipation (according to modified Rome criteria) for more than 50% of their treatment weeks. No treatment-related safety differences were observed between the PEG and placebo groups during the study, with the exception of GI complaints (39.7% PEG-treated patients *vs* 25% placebo-treated patients; *P*= 0.015). This difference was observed due to abdominal distension, diarrhea, loose stools, flatulence and nausea, which are considered usual effects of laxative use.^48^ In addition, a retrospective study of institutional patients with chronic constipation reported good long-term results for up to 12 months,^49^ and for up to 5 months in a randomized prospective study in adult^50^ and pediatric constipated patients.^51^

Stimulant laxatives act via the lumen to alter electrolyte transport and increase intraluminal fluid secretion; when in contact with the mucosa they indirectly stimulate sensory nerve endings, thereby stimulating propulsion.^41^,^42^ Based on older literature, stimulant laxatives are more effective than placebo.^52^ A recent 4-week placebo-controlled trial with picosulfate 10 mg daily showed that this laxative was superior to placebo in increasing the number of (complete) spontaneous bowel movements, and in improving symptoms of straining and some aspects of quality of life. However, nearly 50% of the patients down-titrated the dose.^53^ Stimulant laxatives (especially bisacodyl) are traditionally used as rescue therapy in recent therapeutic trials in chronic constipation, suggesting that they are considered to have superior efficacy, although comparative trials with other agents are lacking.^54^,^55^ There is evidence that stimulant laxatives may cause abdominal pain.^56^ Early concerns of a possible link between chronic anthraquinone use, colonic inertia, and colon cancer have not been substantiated.^37^,^57^

In summary, a wide variety of laxatives are available, many of which are effective and well-tolerated in most constipated patients. However, they are not effective in all patients, and for some, the mode of action or dosage schedule is unacceptable and leads to patient dissatisfaction.^2^ In Table 4, we have summarized the available data on the use of dietary fiber, laxatives, and treatment of chronic constipation.^40^,^55^,^58^ Although the levels of evidence and grading of the recommendations varies between the meta-analyzes, in general:

**Table 4:** Evidence-based review of treatments for constipation

Recommendation			
Agent/procedure	Ramkumar & Rao (2001)^58^	ACG chronic constipation task Force (2005)^55^	American Society of Colon and Rectal Surgeons (2007)^40^
Dietary fiber/bulking agent	1C	B (psyllium)	B (fiber/psyllium)
Osmotic laxatives	1B	A (PEG and lactulose)	A (PEG) B (lactulose)
Diphenylmethanes†, anthraquinones	2B	Insufficient evidence (Grade B)	C
Stool softeners	–	Insufficient evidence (Grade B)	C
Lubiprostone*	–	–	A
Biofeedback therapy‡ (selected cases)	1B	Insufficient evidence (Grade C)	B
Surgery (severe colonic inertia)	2B	–	B

* Not available in Europe with the exception of Switzerland.

† Recent controlled trial shows efficacy of sodium picosulfate in patients with chronic constipation (Rome II).^53^

‡ Recent controlled trials indicate an established efficacy for biofeedback in patients with disordered defection.^27^–^29^

1. Grade A recommendations are supported by good evidence.
2. Grade B recommendations are supported by moderate evidence.
3. Grade C recommendations are only supported by poor evidence.
4. Grade 1 recommendations are supported by one or more randomized clinical trials.
5. Grade 2 recommendations are supported by one or more well-designed cohort or case-controlled studies.
6. Grade 3 recommendations are supported by expert opinion based on clinical experience.

Recent controlled studies have established the efficacy of biofeedback in the management of chronic constipation in those with defecatory disorders, but the efficacy seems less in those with slow-transit constipation.^27^–^29^ This establishes biofeedback as the treatment of choice for constipation with defecation disorders. However, recognizing and diagnosing defecatory disorders is beyond the scope of primary care practice, and is usually only done by gastroenterologists. Moreover, this therapy requires a patient who understands the concept and aim of the biofeedback process, and a skilled and motivated physiotherapist. Availability of experienced therapists and reimbursement of biofeedback is problematic in most parts of Europe.

### New pharmacological treatments for constipation

#### Currently available treatments

##### Lubiprostone

Chloride channels play an important role in fluid transport and the maintenance of cell volume and pH in a variety of tissue and cell types, and in particular, in intestinal epithelial cells.^59^ Nine separate channels have been identified, of which the CIC-2 channel is of particular interest; when it is activated, the secretion of intestinal fluid is promoted.^60^ The secretion of fluids into the GI tract improves stool consistency and may contribute to normal transit.

Lubiprostone activates chloride channels to increase intestinal fluid secretion.^61^ However, its exact mechanism is unclear and may involve type-2 chloride channels, cystic fibrosis transmembrane conductance regulator chloride channels and/or G-protein-coupled prostaglandin receptors.^62^ It has been shown to significantly increase bowel movement frequency [5.89 spontaneous bowel movement (SBM)/week *vs* 3.99, *P*<0.0001] and relieve other constipation-related symptoms compared with placebo.^61^,^63^ It was approved by the FDA in 2006 for the treatment of chronic idiopathic constipation in adults, and subsequently for constipation-predominant IBS (IBS-C) in 2008, but, apart from Switzerland,^64^ it is not approved in Europe, as the Marketing Authorization Application was withdrawn in September 2009 as a result of a strategic decision by Sucampo Pharma Europe, Ltd.

##### 5-HT_4_ agonists

As has already been discussed, serotonin is a critical component in the regulation of gut motility, visceral sensitivity, and intestinal secretion, acting via serotonin 5-HT_4_ receptors, which are expressed mainly by ENS interneurones. It is logical, therefore, to target 5-HT_4_ receptors in developing treatments for constipation. 5-HT_4_ receptor agonists stimulate 5-HT_4_ receptors on ENS interneurones to enhance the peristaltic reflex and have been shown to be effective in the treatment of chronic constipation.^65^ However, the poor selectivity of the early 5-HT_4_ receptor agonists, such as cisapride and tegaserod, has affected their risk : benefit profile and has ultimately restricted their clinical use. Cisapride, a member of the substituted benzamide family, is a partial 5-HT_4_ receptor agonist that was widely used for the treatment of gastro-esophageal reflux and dyspepsia before its withdrawal from the market in July 2000. It was associated with rare dose-dependent cardiac events, including lengthening of the QT interval, syncope, and ventricular arrhythmia in patients with predisposing conditions.^66^ This effect is now believed to be caused by its interaction with the cardiac hERG potassium channel.^67^ Tegaserod, an aminoguanidine indole, is a 5-HT_4_/5-HT_1_ receptor partial agonist, a 5-HT_2_ receptor antagonist, and has been shown to inhibit dopamine and noradrenaline transporters.^68^ It was previously approved in the USA (but not in Europe, apart from Switzerland), but was withdrawn in March 2009 because of a possible increased risk of cardiovascular adverse events (AEs) and is now only available for emergency use. Although the mechanisms underlying these adverse effects are not clear, they are unlikely to be related to 5-HT_4_ effects.^69^,^70^ More recently, a number of new 5-HT_4_ receptor agonists, with better selectivity profiles, have been developed.

##### Prucalopride

Prucalopride is highly selective for the 5-HT_4_ receptor, unlike cisapride, displaying at least 150-fold selectivity for its therapeutic target receptor.^68^,^71^ Early studies demonstrated that it decreased colonic transit time in normal and constipated subjects.^72^,^73^ Three large randomized Phase III controlled trials with a total of 1977 patients (1750 female and 227 male) with severe chronic constipation (defined as ≤2 SCBM/week for a minimum of 6 months with either very hard or hard stools, sensation of incomplete evacuation or straining during defecation for at least 25% of the time) confirmed that, averaged over 12 weeks, bowel function (measured as an increase of ≥1 SBM/week) was significantly improved in up to 69% of patients receiving the recommended dose of 2 mg prucalopride, with a median time of 2.5 h to first SBM.^74^–^76^ Transit studies showed that prucalopride was found to accelerate gastric emptying, small bowel transit, overall colon transit, and ascending colonic emptying in patients with functional constipation.^73^ Phase III trials have also confirmed that, in addition to improving bowel function, prucalopride significantly improves patient treatment satisfaction and quality of life, and alleviates a broad spectrum of constipation-related symptoms, including bloating, abdominal discomfort, and defecation urge with inability to evacuate.^74^–^77^ The most common adverse reactions (headache, nausea, diarrhea and abdominal pain) were mild and usually disappeared after the first day of treatment.^78^ Safety data from two randomized Phase I cross-over trials showed no clinically significant results from 24-h Holter monitoring. No difference was observed in the mean QT values corrected according to Fridericia's method (QTcF) when comparing prucalopride with placebo, and no QTcF values exceeded 500 ms or increased >60 ms during the treatment periods.^79^ These data indicate that there was no increased risk of drug-induced QT prolongation in this study.^79^ A randomized Phase II dose-escalating safety study conducted in 89 elderly patients aged ≥65 years old with constipation (87.7% of whom had a history of cardiovascular disease), also showed no clinically relevant differences in QT or QTcF time intervals between prucalopride and placebo, suggesting there is no specific cardiovascular safety concern in the elderly.^80^ Results from a long-term (24 months) open-label extension study show that treatment satisfaction is maintained with prolonged use; no safety signals were recorded in this study.^81^ Prucalopride has also been shown to be effective in the elderly 82 and patients with opioid-induced constipation.^83^

In October 2009, prucalopride received EU approval for the treatment of chronic constipation in women in whom laxatives fail to provide adequate relief, and now represents a new therapeutic option in the management of this condition. The therapeutic potential in hypomotile disorders of both the upper and lower GI tract, and the different pharmacological mode of action, might be the reason why the regulatory authorities have classified prucalopride in a separate class to laxatives (WHO ATC classification *A03AE, drugs for functional bowel disorders – acting on serotonin receptors*).

#### Experimental treatments

##### Opioid antagonists

Three mu-opioid antagonists (naloxone, methylnaltrexone and alvimopan) are currently under evaluation for the treatment of opiate-induced constipation 84,85 and postoperative ileus.^86^ Although endogenous opioids may play a role in modulating GI function,^87^ early reports suggested that opioid antagonists are not effective in idiopathic constipation.^88^

##### Linaclotide

Linaclotide is an agonist of guanylate cyclase-C receptors, which stimulates intestinal fluid secretion and transit. In early studies, it has been found to increase bowel movement frequency and loosen stool consistency.^89^ A recently published dose range-finding study and results from two Phase III trials in 1272 patients with chronic constipation, show that linaclotide significantly improved bowel function (measured as ≥3 complete SBMs (SCBM) per week, with an increase of ≥1 from baseline for ≥9 of 12 weeks) in up to approximately 20% of patients.^90^ The median time to first SBM was 21.9 h (150 *μ*g).^91^ Furthermore, abdominal symptoms, global measures of constipation and quality of life were also significantly improved 91,92 and there was no evidence of rebound constipation upon treatment cessation.^93^ The most common AEs were GI-related, of which diarrhea had the highest incidence.^91^ Linaclotide is currently not licensed for use in the EU.

##### Other 5-HT_4_ agonists

Other enterokinetic agents in development include the 5-HT_4_ receptor agonists TD-5108 (Phase II),^94^ and ATI-7505 (Phase II).^95^ A number of other prokinetic 5-HT_4_ receptor agonists have been developed for GI disorders, which are of considerable therapeutic interest but are in the early stages of development.

## Review of currently available guidelines, recommendations and algorithms

A number of groups have provided recommendations for the diagnosis and treatment of constipation;^32^,^35^,^40^,^96^,^97^ however, no standardized treatment guidelines have gained acceptance in general medical practice. Although the evidence for a number of interventions (including modifications to diet and lifestyle) is weak or contradictory, all the guidelines recommend that these be tried before pharmacological intervention. In general, where treatment pathways are recommended, the sequence is:

1. Exclude other pathologies and secondary causes.^35^,^40^,^55^,^96^,^97^
2. Begin treatment with dietary and lifestyle adjustments.^35^,^40^,^55^,^96^,^97^
3. Move to osmotic laxatives, stool softeners and bulk-forming agents – there is no consensus on the order in which these should be tried.^35^,^96^,^97^
4. Move to stimulant laxatives, suppositories and/or enemas^96^,^97^– some guidelines recommend medical supervision at this stage.^97^
5. Surgery should be used as a last resort or to treat identified disorders that require surgical correction.^40^,^97^

Although prokinetic agents feature in the two sets of US guidelines (Grade A recommendation),^40^,^55^ these are now out of date. Tegaserod has now been limited to emergency use in the US and has not received licensing approval in the EU. Prucalopride has recently received EU approval for the treatment of chronic constipation in women in whom laxatives fail to provide adequate relief; this is not mentioned in the guidelines.

Once organic disorders and obstructions have been excluded, a functional bowel disorder is the most likely explanation for the constipation. Most patients with chronic constipation report minimal abdominal bloating or discomfort associated with their other symptoms of chronic constipation; however, in some patients, as symptoms often overlap, it may be difficult to distinguish chronic constipation and IBS-C.^55^ The American College of Gastroenterology (ACG) Chronic Constipation Task Force defined IBS-C as clinically important abdominal discomfort associated with symptoms of constipation.^61^ National Institute for Clinical Excellence (NICE) guidelines for IBS indicate a positive diagnosis only if the person has abdominal pain/discomfort that is either relieved by defecation, or associated with altered bowel frequency or stool form, and at least two of the following: altered stool passage; abdominal bloating, distension, tension, or hardness, symptoms made worse by eating and passage of mucus.^98^

**Figure 2:**
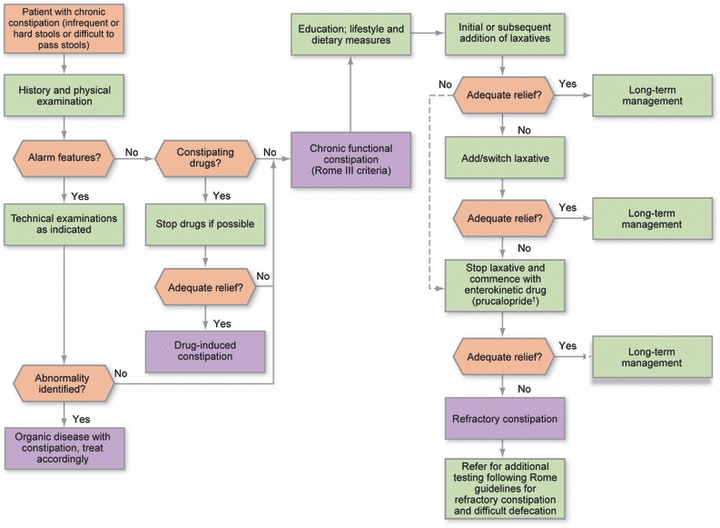
Enterokinetic treatment algorithm. Once idiopathic chronic constipation has been identified (Rome III); and education, lifestyle and dietary measures; and treatment with laxatives (response evaluable after 2–4 weeks) have failed to provide adequate relief, an enterokinetic agent can be commenced (response to prucalopride evaluable after 4–12 weeks). If constipation symptoms are still refractory to pharmacological treatment, patients should be referred for physiological testing as outlined in the published Rome algorithm for refractive constipation and difficult defecation. ^†^2 or 1 mg day^−1^ if the patient is >65 years.

**Figure 3:**
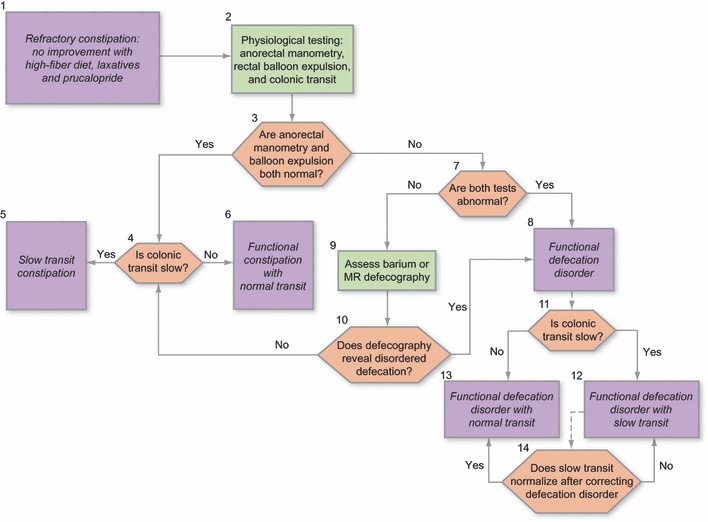
Refractory constipation and difficult defecation. (1) Patients who fulfill the criteria for functional constipation and those who have not improved with an increase in dietary fiber and the use of simple laxatives, and with no alarm features, often warrant further physiological assessment. (2) The three key physiological investigations are anorectal manometry, the balloon expulsion test, and a colonic transit study. (3, 4) If both anorectal manometry and balloon expulsion are normal, the results of colonic transit testing enable characterization of the disorder as functional constipation with slow (5) or normal transit (6). (7, 8) If both manometry and the rectal balloon expulsion test are abnormal, this is sufficient to diagnose a functional defecation disorder. (9) If only one of the anorectal manometry and balloon expulsion is abnormal, further testing using barium or magnetic resonance defecography may be used to confirm or exclude the diagnosis. (10) If defecography reveals features of disordered defecation, a diagnosis of a functional defecation disorder can be made. (8) If defecography is not abnormal, then the patient does not fulfill criteria for the diagnosis of a functional defecation disorder; further diagnosis then depends on the presence or absence of colonic transit delay (see above 4–6). (11–13) Treatment of choice for disordered defecation is biofeedback. If there is no adequate response to therapy, further investigation may be considered at this point. The presence of a functional defecation disorder does not exclude the diagnosis of slow colonic transit. Thus, depending on the results of the colonic transit study, the patient can be characterized as suffering from a functional defecation disorder with slow (12) or normal colonic transit. (13, 14) Slow colonic transit may result from a defecation disorder. If it is felt appropriate to distinguish between the two possibilities, the colonic transit evaluation may be repeated after correction of the defecation disorder. If transit normalizes, the presumption is that the delay was secondary to the defecation disorder; if not, the delayed colonic transit is presumed to be a comorbid condition, which may require therapy if there is no clinical improvement with the treatment of functional defecation disorder. This figure has been adapted by permission from Macmillan Publishers Ltd: The American Journal of Gastroenterology,^36^ copyright (2010).

As previously mentioned, guidelines and algorithms for the management and treatment of chronic constipation have not taken into account more recent therapeutic developments. Although a new set of Rome Foundation diagnostic algorithms covering the diagnosis and management of FGIDs including chronic constipation^35^ and refractory constipation^36^ have been recently published, newer agents have not been included. According to these guidelines, patients with constipation that is refractory to a high-fiber diet and traditional laxatives should be referred for physiological testing, such as anorectal manometry, rectal balloon expulsion, and colon transit. Now, with the recent availability of prokinetic agents such as prucalopride, an additional therapeutic step can be added to these existing guidelines. Once idiopathic chronic constipation has been identified, and IBS and secondary constipation have been excluded, empirical treatment with osmotic and/or stimulant laxatives should be employed. Following this, if patients still experience continuous symptoms (e.g., bloating, abdominal discomfort and incomplete bowel movements), a prokinetic agent such as prucalopride could be considered (Fig. 2). If constipation symptoms are refractory to pharmacological treatment, patients should be referred for physiological testing as outlined in the published Rome algorithm for refractive constipation and difficult defecation (Fig. 3).^36^ Patients should only be referred for surgery following colon transit testing without, and then with, laxatives.

## Conclusions

Constipation is common and for some it can be chronic, where symptoms can be severe and can significantly affect a patient's quality of life. Although many laxative treatments are available, either OTC or by prescription, patients may often need additional treatment to achieve optimal symptom relief. As evidence for the effectiveness for many of the older laxatives is limited and there are relatively few guidelines on the management of this condition, treatment is often empirically-based. If diet, lifestyle measures, and traditional laxative therapies fail to provide adequate relief, the use of a motility agent offers a novel mechanism of action with therapeutic benefit. The 5-HT_4_ agonist prucalopride, approved in the EU, increases colonic motility and is a valuable clinical option for the patient who is dissatisfied or incompletely treated by laxatives.
